# Prevalence and Risk Factors for HTLV-1/2 Infection inRiverside and Rural Populations of the State of Pará

**DOI:** 10.3390/v14102262

**Published:** 2022-10-15

**Authors:** Aline Cecy Rocha de Lima, Felipe Teixeira Lopes, Vanessa de Oliveira Freitas, Michele Nascimento Assad, Renata Santos de Sousa, Janete Silvana Souza Gonçalves, Jayanne Lílian Carvalho Gomes, Bernardo Cintra dos Santos, Carlos Neandro Cordeiro Lima, Isabella Nogueira Abreu, Wandrey Roberto dos Santos Brito, Keise Adrielle Santos Pereira, Maria Karoliny da Silva Torres, Sandra Souza Lima, Cíntia Yolette Urbano Aben-Athar, João Farias Guerreiro, Izaura M. V. Cayres Vallinoto, Antonio Carlos Rosário Vallinoto, Rosimar Neris Martins Feitosa

**Affiliations:** 1 Laboratório de Virologia, Universidade Federal do Pará, Belém 66075110, Brazil; 2 Programa de Pós-Graduação em Biologia de Agentes Infecciosos e Parasitários, Instituto de Ciências Biológicas, Universidade Federal do Pará, Belém 66075110, Brazil; 3 Laboratório de Genética Humana e Médica, Universidade Federal do Pará, Belém 66075110, Brazil

**Keywords:** HTLV 1/2, riparian, rural area

## Abstract

Human T-lymphotropic viruses 1 and 2 (HTLV-1 and HTLV-2) infection has been described in several Amazonian populations; however, there is still a lack of data on the prevalence of the virus in riparian populations living in rural areas of the state of Pará. The present study aimed to evaluate the prevalence of HTLV-1/2 infection in four riverine communities and one rural area in the state of Pará and to describe the possible risk factors for infection. A total of 907 individuals responded to an epidemiological survey and gave blood samples collected for anti-HTLV-1/2 antibodies by immunoenzymatic assay (EIA). The serum-reactive samples were subjected to confirmation by an in-line assay (Inno-Lia) and by proviral DNA screening using real-time PCR (qPCR). The total prevalence was 0.8% (7/907) for HTLV-1/2 (CI: 0.2−1.3%), with 0.66% HTLV-1 and 0.11% HTLV-2. The prevalence by sex was 0.7% in women (4/565) and 0.9% in men (3/342). Among seropositive patients, 83.3% (5/7) reported being sexually active, and 57.1% (4/7) reported not having the habit of using condoms during their sexual relations. Intrafamily infection was also observed. The results reinforce the need for public policies to prevent and block the spread of HTLV, especially in riparian communities that are subject to difficulties in accessing the Unified Health System (Sistema Único de Saúde/SUS) because infected individuals need clinical monitoring for surveillance and early diagnosis of symptoms associated with HTLV-1.

## 1. Introduction

The human T-lymphotropic virus (HTLV), belonging to the *Retroviridae* family, was first described in 1980 [1] and has since been reported in different populations worldwide with heterogeneous prevalence [2] and associated with different lymphoproliferative diseases (leukaemia/adult T-cell lymphoma-ATLL) and inflammatory diseases, such as HTLV-1-associated myelopathy (HAM), uveitis, arthritis, polymyositis and others [3,4,5,6,7,8].

It is estimated that Brazil has the highest number of absolute cases of HTLV infection, with approximately 800,000 to 2.5 million people infected [9,10], and epidemiological studies have already demonstrated the presence of this virus in several areas such as São Paulo [11], Santa Catarina [12], Salvador [13], and Pará [14], as well as other states. Maranhão, Bahia and Pará have high prevalence rates of the virus, as indicated in studies performed in blood donors, in pregnant women or in the general population [15,16].

Among the main groups described in the literature and related to HTLV infection in the Amazon region of Pará are blood donors, people living with HIV, Japanese people and indigenous people [17,18,19,20]. It is worth noting that in the state of Pará, HTLV-2 was first detected outside indigenous populations in 1998, and both viruses were found in blood donors, drawing attention to the need for blood centres to test for HTLV 1/2 [16].

Although HTLV-1/2 infection has been described in some populations of the Brazilian Amazon region, especially in indigenous and urban communities [14,15,16,17,18,19,20], there is still a lack of data that accurately characterize the prevalence of infection in riparian communities and rural areas in the Pará region, which have limited access to public health services [21,22]. Therefore, knowing the prevalence of HTLV-1/2 in riparian and rural populations is of paramount importance for a more accurate description of the geographical distribution of the infection and the risk factors for spreading the virus to design control measures and public health policies for prevention and control, since to date there is no effective treatment to combat the virus and the main associated diseases or a protective vaccine [23].

Thus, the objective of this study was to describe the prevalence of HTLV-1/2 epidemiological and behavioural aspects of risk for exposure to the virus in rural and riverine communities located in the state of Pará, Brazil.

## 2. Materials and Methods

**Figure 1:**
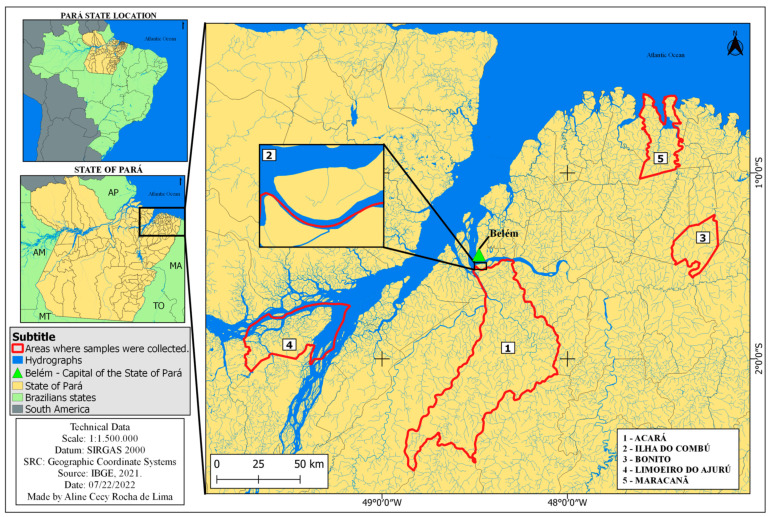
Geographic locations of collection areas for riverine and rural populations in the state of Pará.

From June 2020 to August 2022, five riverine and rural communities located in the state of Pará were visited, and a socio-epidemiological questionnaire about behavioural risk factors for HTLV-1/2 infection was administered to residents. A total of 907 individuals participated in the study, including riverine residents of Combú Island (*n* = 357; estimated population of 985 inhabitants), Acará (*n* = 55; estimated population of 500 inhabitants), Limoeiro do Ajurú (*n* = 111; estimated population of 4800 inhabitants) and the rural areas of the municipalities of Bonito (*n* = 267; estimated population of 6000 inhabitants) and Maracanã (*n* = 118; estimated population of 325 inhabitants) (Figure 1). Individuals of both sexes and of different ages, randomly selected (spontaneous demand), participated in the study.

A blood sample (5 mL) was collected from each participant for serological and molecular biology tests. Aliquots of plasma and leukocytes were separated by centrifugation (8000 rpm for 15 min) and stored at −20 °C until use.

### 2.1. Ethical Aspects

The present study was approved by the National Research Ethics Committee (Conselho Nacional de Ética em Pesquisa/CONEP) (CAAE: 27290619.2.0000.0018), following resolution 466/12 of the Ministry of Health, responsible for regulating any research involving human beings.

### 2.2. Serological Screening

The detection of anti-HTLV-1/2 antibodies was performed using an ELISA kit (Murex HTLV-I+II, DiaSorin, Dartford, UK) following the protocol suggested by the manufacturer. All samples considered reactive or indeterminate in this test were subjected to confirmatory tests (INNO-LIA and/or real-time PCR).

### 2.3. Line Immunoassay (LIA)

The INNO-LIA^®^ HTLV I/II Score (Fujirebio, Japan) was used as a method for confirmation and differentiation of viral types, following the manufacturer’s protocol.

### 2.4. DNA Extraction

The analysis of HTLV proviral DNA was performed using 200 µL of whole blood, which was subjected to DNA extraction with the aid of the QiaAmp DNA mini kit (Qiagen, Hilden, Germany) according to the protocol defined by the manufacturer. Prior to the amplification of the targets, the samples were quantified using the Qubit 2.0 fluorometer (Invitrogen, Waltham, MA, USA).

### 2.5. Real-Time PCR

Molecular confirmation of HTLV infection was performed by a single-plex real-time PCR using the TaqMan system (Applied Biosystems, Foster City, CA, USA) on the Applied Biosystems StepOne Plus Real Time PCR platform with three target sequences: the albumin gene, as endogenous control, and non-homologous regions of the HTLV-1 *pol* (186 bp) and HTLV-2 *tax* (75 bp) genes as molecular markers of the virus, adapted from [24]. 

In each reaction, 12.5 µL of TaqMan Universal PCR Master Mix (2X) (Applied Biosystems, Foster City, CA, USA), 6.0 µL of ultrapure water, 0.5 µL of each primer (10 pmol), 0.5 µL of each probe (5 mM) and 5.0 µL of DNA (50 ng) were used, resulting in a total volume of 25 µL. The temperature cycles used were 95 °C for 10 min, followed by 45 cycles of 95 °C for 15 s and 60 °C for 1 min.

The following primers were used in the reactions: 5′-CCCTACAATCCAACCAGCTCAG-3′ (HTLV-1F), 5′-GTGGTGAAGCTGCCATCGGGTTTT-3′ (HTLV-1R), 5′-CGATTGTGTACAGGCCGATTG-3′ (HTLV-2F), 5′-CAGGAGGGCATGTCGATGTAG-3′ (HTLV-2R), 5′-GCTGTCATCTCTTGTGGGCTGT-3′ (Albumin F), and 5′-AAACTCATGGGAGCTGCTGGTT-3′ (Albumin R). The probe sequences were as follows: FAM-5′-CTTTACTGACAAACCCGACCTACCCATGGA-3′-MGB (HTLV-1), FAM-5′-TGTCCCGTCTCAGGTGGTCTATGTTCCA-3′-MGB (HTLV-2) and FAM-5′-CCTGTCATGCCCACACAAATCTC-3′-MGB (Albumin).

### 2.6. Statistical Analysis

The data obtained based on the questionnaires answered by the participants were added to the Epi-Info 7.2 database. Statistical analyses were performed using BioEstat 5.3 [25]. All variables studied were analysed using descriptive statistics, and the estimated prevalence was analysed using point estimators and confidence intervals (95% confidence intervals (CIs)). To identify the epidemiological characteristics associated with HTLV infection, the chi-square and Fisher’s exact tests were applied, adopting a significance level of 95% (*p* < 0.05).

## 3. Results

The serological investigation showed a prevalence of anti-HTLV-1/2 antibodies of 0.8% (7/907) (CI: 0.2–1.3%) for the total of riparian and rural populations evaluated in the present study, with six individuals infected by HTLV-1 (0.7%) and one infected with HTLV-2 (0.1%). The analysis by community revealed seroprevalences of 0.3% (1/357) on Combú Island, 1.8% (2/111) in Limoeiro do Ajurú and 3.3% (4/118) in Maracanã. No reactive samples were found in the Acará and Bonito populations. Among the seropositive samples in the ELISA test, five were confirmed by INNO-LIA and by qPCR as HTLV-1. The only sample positive for HTLV-2 was confirmed only by INNO-LIA, and amplification was not detected in the qPCR (Table 1).

Regarding the risks of exposure to HTLV-1/2 infection among seropositive volunteers, it was observed that 83.3% (5/7) reported being sexually active, 100% (7/7) reported not having tattoos or piercings, not having received blood transfusion, and were breastfed during childhood. In addition, 57.1% (4/7) reported not having the habit of using condoms in their sexual relations, 83.3% (5/7) never received a diagnosis of sexually transmitted infections (STIs), and 85.7% (6/7) reported never having sex in exchange for money (Table 2).

The two individuals who had a positive diagnosis, residents of the municipality of Limoeiro do Ajurú, belonged to the same nuclear family, with individual #94 being the mother of individual #63, thus identifying a possible occurrence of intrafamily infection by mother-to-child transmission via the vertical route (delivery) or even through breastfeeding. The difficulty of access to the community and the nonadherence to participation in the study prevented the testing of other members of this family unit, such as the spouse of individual #94.

## 4. Discussion

The seroprevalence of HTLV-1/2 in the state of Pará has already been described heterogeneously in different groups and communities [26,27,28]. The present study found a global HTLV-1/2 prevalence of 0.8% (0.66% for HTLV-1 and 0.11% for HTLV-2), and these infections were confirmed for the first time in Combú Island (HTLV-2), Limoeiro do Ajurú (HTLV-1) and Maracanã (HTLV-1). These results are in agreement with what has been described in other riparian communities that reported rates ranging from 0 to 1.6% [29]. However, seroprevalence is usually higher in rural areas, ranging from 2.3 to 2.7% [19,20,21,22,23,24,25,26,27,28,29,30]. The differences in prevalence observed in the communities studied herein reflect the heterogeneity of the distribution of HTLV-1/2 infection, as already described in other human populations [31], which may be the result of different risk factors and modes of transmission.

A low prevalence of HTLV-1 and HTLV-2 among the quilombola population has been described, ranging from 0.11 to 2.6% and 0.34 to 1.06%, respectively [32,33]. In the urban region of Belém, the rates are usually higher, ranging from 0.19 to 2% [14,15,16,17,18,19,20,21,22,23,24,25,26,27,28,29,30,31,32,33,34], as well as in a group of people living with HIV [35], blood donors [17], and sex workers [36], while in pregnant women, this prevalence is considerably lower [16]. In the Pará region, a high prevalence of HTLV-2 is usually described among indigenous populations because they are closed communities [37,38,39].

The main limitation of our study was the sample size obtained from the communities, a fact resulting from the health restrictions imposed by the COVID-19 pandemic, which made it difficult to access a more representative sample of the populations studied and, possibly, a more accurate estimation of HTLV-1/2 infection.

Most participants in this study had a low level of education, which may be related to their level of knowledge about ways to contract STIs [40]. During the application of the questionnaire to rural and riverine populations, it was observed that many did not understand what was being asked, and pauses were necessary for explanations on the subject. For this reason, although a large portion of the individuals were positive for HTLV-1/2 and reported that they were never diagnosed with other STIs, these statements cannot be considered reliable because many did not understand the questions.

The difficulty of access to urban areas, where medical care can be found more easily, is also an unfavourable condition for access to health in general [41,42]. This is all associated with the fact that HTLV-1/2 usually evolves silently [43] and may further contribute to late diagnoses in this population.

Although the results are not statistically significant, the infection rates for HTLV-1/2 were higher for females, which is in agreement with what has been reported in previous studies [38,39,40,41,42,43,44,45,46]. This can be explained by the fact that: (i) the number of women investigated was higher than that of men, or (ii) the transmission occurs more frequently from men to women due to the female genitourinary characteristics that facilitate the transmission of the virus and the presence of infected cells in semen [47]. 

Most infected individuals reported being sexually active and not having the habit of using condoms during sexual intercourse, a fact that may explain the infection in these individuals, since sexual intercourse is considered the main means of HTLV infection [48,49]. In addition, the non use of condoms has been reported as a bad habit among riverine populations [50] and may be related to the fact that they are commonly in long-term relationships with the same partners and do not deem it necessary to use condoms [51].

The Brazilian Protocol of Sexually Transmitted Infections 2020: Infection by the Human T-lymphotropic Virus describes breastfeeding as a route of viral transmission [52,53]. In the present study, all seropositive patients reported having been breastfed during childhood, and two were identified as mothers and children residing in Limoeiro do Ajurú, demonstrating that in addition to the non use of condoms, vertical transmission can be considered one of the forms of transmission within this population.

According to the Brazilian Institute of Geography and Research (*Instituto Brasileiro de Geografia e Pesquisa/IBGE*) [54], the municipality of Maracanã, which reported a high prevalence rate for HTLV-1 in this study, was once inhabited by an indigenous village known by the same name. However, the presence of HTLV-1 as a result of the presence of miscegenation with Afro-descendants cannot be ruled out, and the same aspect is valid for the community of Limoeiro do Ajurú. This historical fact, associated with the occurrence of HTLV-1/2 infection in different indigenous peoples in Brazil [37,38] and the interethnic miscegenation process of the Amazonian population [55], suggests that the presence of HTLV-1 and HTLV-2 in the rural and riverside communities may reflect the Indigenous and African contributions to the ethnic composition of the current Amazon populations, which may have influenced the transmission of the virus in that location. However, more studies are needed to better understand this prevalence.

Among the study participants who were diagnosed with HTLV-1/2, it was possible to perform the second collection of only two individuals, who were confirmed as positive by means of real-time PCR. Because they are riparian populations, some communities where these individuals reside are located in areas of difficult access and communication; however, the team of the Virology Laboratory of the Federal University of UFPA is still seeking ways to contact positive individuals again for the performance of extended evaluation of intrafamily transmission.

## 5. Conclusions

The frequency of HTLV-1/2 detected in the populations of the present study shows variations in prevalence, reinforcing the heterogeneity of the infection observed in different geographic areas. Riparian people are vulnerable populations due to socioeconomic factors such as a lack of education and access to health. In addition, the fact that HTLV is still neglected in Brazil has further contributed to the difficulty of detecting new cases. Public policies and strategies that can act in the control, prevention, and promotion of information and allow additional investigation of intrafamily viral infection are considered the best options to support these populations.

## Figures and Tables

**Table 1:** Demographic data of HTLV-1 positive patients and results of tests performed to confirm the diagnosis of HTLV 1/2 infection.

ELISA							Real-Time PCR			INNO-LIA							
	Location	Sex	Age	anti-HTLV 1/2	OD	*pol*-1	*tax*-2	Ct	Result	gag p19 I/II	gag p24 I/II	env gp46 I/II	env gp21 I/II	gag p19/I	env gp 46/I	env gp 46/II	Result
**Individual 06**	Combú Island	F	72	Reactive	>2.0	ND	ND	ND	ND	+	+	+	+	-	-	+	**HTLV-2**
**Individual 16**	Maracanã	F	69	Reactive	>2.0	Detectable	ND	29.3	HTLV-1	+	+	+	+	+	+	-	**HTLV-1**
**Individual 90**	Maracanã	M	49	Reactive	>2.0	Detectable	ND	27.7	HTLV-1	+	+	+	+	+	+	-	**HTLV-1**
**Individual 116**	Maracanã	F	49	Reactive	>2.0	Detectable	ND	30.1	HTLV-1	+	+	+	+	+	+	-	**HTLV-1**
**Individual 117**	Maracanã	M	53	Reactive	>2.0	Detectable	ND	30.8	HTLV-1	+	+	+	+	+	+	-	**HTLV-1**
**Individual 63**	Limoeiro do Ajurú	M	14	Reactive	>2.0	Detectable	ND	34.9	HTLV-1	+	-	+	+	-	+	-	**HTLV-1**
**Individual 94**	Limoeiro do Ajurú	F	43	Reactive	>2.00	Detectable	ND	26.2	HTLV-1	Not tested	Not tested	Not tested	Not tested	Not tested	Not tested	Not tested	**HTLV-1**

M: Male; F: Female; ND: Not detectable; +: Positive; -: Negative. Ct: Cycle threshold; OD: Optical density. The socio-epidemiological characteristics of the 907 study participants are described in Table 2. Among the individuals considered positive for HTLV-1/2, 57.1% (4/7) were female, had incomplete primary educations and family income equivalent to one minimum wage salary. Regarding the age group of the seven seropositive individuals, a single individual (1/7) was between 12 and 18 years of age (14.3%), four were 19 to 59 years (57.1%) and two were over age 60 (28.6%).

**Table 2:** Risk characteristics for contracting HTLV 1/2 infection in rural and riverine populations of the state of Pará.

Risk Factors	Population Studied for HTLV 1/2			*p* **
	Total *n* (%)	Positive *n* (%)	Negative *n* (%)	
**Sex**				
Female	565 (62.3)	4 (57.1)	561 (62.3)	1.000
Male	342 (37.7)	3 (42.9)	339 (37.7)	
**Age**				
7–11	23 (2.6)	0	23 (2.6)	
12–18	54 (6.0)	1 (14.3)	53 (6.0)	0.5149
19–59	694 (78.0)	4 (57.1)	691 (78.2)	
≥60	119 (13.4)	2 (28.6)	117 (13.2)	
Not informed *	17	0	16	
**Sexually active**				
Yes	585 (80.7)	5 (83.3)	580 (80.7)	1.000
No	140 (19.3)	1 (16.7)	139 (19.3)	
Not informed	182	1	181	
**Tattoo**				
Yes	90 (10.7)	0	90 (10.6)	1.000
No	770 (89.3)	7 (100)	763 (89.4)	
Not informed	47	0	47	
**Pierced**				
Yes	24 (2.8)	0	24 (2.8)	1.000
No	827 (97.8)	7 (100)	820 (97.2)	
Not informed	56	0	56	
**Blood transfusion**				
Yes	58 (6.8)	0	58 (6.9)	1.000
No	790 (93.2)	7 (100)	783 (93.1)	
Not informed	59	0	59	
**Use of condoms**				
Yes	219 (27.9)	3 (42.9)	216 (27.8)	
No	422 (53.8)	4 (57.1)	418 (53.7)	0.2520
Sometimes	144 (18.3)	0	144 (18.5)	
Not informed	112	0	112	
**Practised sex for money**				
Yes	42 (5.5)	0	42 (5.5)	1.000
No	726 (94.5)	6 (100)	720 (94.5)	
Not informed	139	1	138	
**Diagnosis for STI**				
Yes	25 (6.0)	0	25 (6.1)	
No	345 (83.1)	5 (83.3)	340 (83.1)	0.7202
Does not know	45 (10.9)	1 (16.7)	44 (10.8)	
Not informed	492	1	491	
**Breastfed during childhood**				
Yes	790 (96.1)	7 (100)	783 (96.1)	1.000
No	32 (3.9)	0	32(3.9)	
Not informed	85	0	85	

* Unanswered questions were not considered in the analyses; ** Significance value of *p*.

## Data Availability

The data analysed in this study are included within the paper.
